# The interaction of affective with psychotic processes: A test of the effects of worrying on working memory, jumping to conclusions, and anomalies of experience in patients with persecutory delusions

**DOI:** 10.1016/j.jpsychires.2013.06.016

**Published:** 2013-12

**Authors:** Daniel Freeman, Helen Startup, Graham Dunn, Emma Černis, Gail Wingham, Katherine Pugh, Jacinta Cordwell, David Kingdon

**Affiliations:** a Department of Psychiatry, University of Oxford, Warneford Hospital, Oxford OX3 7JX, UK; b Centre for Biostatistics, Institute of Population Health, Manchester University, Jean McFarlane Building, Oxford Road, Manchester M13 9PL, UK; c Academic Department of Psychiatry, Faculty of Medicine, University of Southampton, College Keep, 4-12 Terminus Terrace, Southampton SO14 3DT, UK

**Keywords:** Delusions, Worry, Paranoia, Hallucinations, Schizophrenia

## Abstract

Worry has traditionally been considered in the study of common emotional disorders such as anxiety and depression, but recent studies indicate that worry may be a causal factor in the occurrence and persistence of persecutory delusions. The effect of worry on processes traditionally associated with psychosis has not been tested. The aim of the study was to examine the short-term effects of a bout of worry on three cognitive processes typically considered markers of psychosis: working memory, jumping to conclusions, and anomalous internal experience. Sixty-seven patients with persecutory delusions in the context of a non-affective psychotic disorder were randomised to a worry induction, a worry reduction, or a neutral control condition. They completed tests of the cognitive processes before and after the randomisation condition. The worry induction procedure led to a significant increase in worry. The induction of worry did not affect working memory or jumping to conclusions, but it did increase a range of mild anomalous experiences including feelings of unreality, perceptual alterations, and temporal disintegration. Worry did not affect the occurrence of hallucinations. The study shows that a period of worry causes a range of subtle odd perceptual disturbances that are known to increase the likelihood of delusions. It demonstrates an interaction between affective and psychotic processes in patients with delusions.

## 1 Introduction

Worry is conventionally studied within the anxiety disorders, but it has recently been given prominence in a theoretical account of persecutory delusions (Freeman, 2007). Worry brings more implausible paranoid ideas to mind, keeps them there, and escalates the distress. There is increasing evidence to support this view. In a national epidemiological survey, individuals reporting concerns of plots to harm them had almost ten times higher odds of reporting worry than individuals without paranoid fears (Freeman et al., 2011). Cross-sectional studies with patients with persecutory delusions have shown that high levels of worry are common, comparable to those seen in generalised anxiety disorder, and that higher levels of worry are associated with more distressing paranoia (Freeman and Garety, 1999, Freeman et al., 2001, Startup et al., 2007, Morrison and Wells, 2007, Bassett et al., 2009, Freeman et al., 2010). Worry is not simply a consequence of paranoid thoughts however. Longitudinal studies have shown that worry predicts the later development and persistence of paranoid fears (Startup et al., 2007, Freeman et al., 2012b), and an experimental study has shown that higher levels of worry predict the occurrence of paranoid thoughts (Freeman et al., 2008). In the strongest tests of the causal role of worry in persecutory delusions, two clinical intervention studies have shown that directly targeting the cognitive style of worry significantly lessens paranoia (Foster et al., 2010, Hepworth et al., 2011). This series of studies indicates that worry in psychosis may well require the level of attention it receives in the anxiety disorders.

Theoretical accounts of psychosis increasingly highlight both affective and cognitive routes to delusions (e.g. Garety et al., 2001, Myin-Germeys et al., 2003, Bentall et al., 2009). This is not meant to imply that affect has no cognitive mechanisms but to make the distinction between what have traditionally been considered as emotional and psychotic processes. Non-affective cognitive disturbance in psychosis has had the greater focus (e.g. Hemsley, 1993, Frith, 1992, Kapur, 2003, Green et al., 2004), but the emotional route to delusions is gaining evidential support (e.g. Lincoln et al., 2010b, Ben-Zeev et al., 2011, Thewissen et al., 2011). The study of worry in psychosis is an example of the latter affective route, since worry is traditionally considered central to the experience of anxiety problems and it is not listed in descriptions of psychosis, but is there an impact of worry on cognitive processes traditionally associated with psychosis? Is there an interaction between emotional and psychotic processes? To answer this question we examined the immediate effects of a bout of worrying on three processes studied in psychosis: working memory, jumping to conclusions, and perceptual anomalies. Meta-analyses have consistently shown that working memory performance is impaired in individuals with a diagnosis of schizophrenia (e.g. Lee and Park, 2005, Forbes et al., 2009), leading to the view that it may be an endophenotype for the diagnosis (e.g. Horan et al., 2008). Jumping to conclusions, a tendency to seek less data before reaching a decision, has been specifically linked with delusional beliefs, and it has been reliably found in patients with psychosis (see Garety et al., 2007, Freeman, 2007, Fine et al., 2007). Particular cognitive dysfunctions in psychosis are considered to produce anomalies of experience (such as perceptual and attentional disturbances, altered experience of self, aberrant salience) that lead to delusional misinterpretations (e.g. Maher, 1988, Kapur, 2003, Uhlhaas and Mishara, 2007); the presence of such anomalies have been repeatedly found in patients with psychosis (e.g. Chapman, 1966, Phillipson and Harris, 1985, Bunney et al., 1999, Parnas et al., 2003) and to be associated with delusional ideation (e.g. Bell et al., 2006, Freeman et al., 2008, Freeman et al., 2010). We therefore studied worry in relation to three processes commonly considered important cognitive markers of psychosis.

Study of a bout of worry in patients with psychosis has not been reported before. However, a bout of worry in non-clinical worriers has been found to be associated with a reduction in working memory capacity (Hayes et al., 2008, Leigh and Hirsch, 2011), and an anxious mood induction has been found to increase the jumping to conclusions bias in a non-clinical sample (Lincoln et al., 2010a), although not in a delusions group (So et al., 2008). Whether a mood induction increases perceptual anomalies is unknown, but levels of anxiety positively correlate with the presence of perceptual distortions (e.g. Bell et al., 2011, Tone et al., 2011). In the current study it was hypothesized that psychotic processes would be exacerbated when patients engage in a period of worry: that worry makes it harder to process information and reason and that it creates a more subjectively odd state. It was predicted that a decrease in working memory, an exacerbation of the jumping to conclusions reasoning style, and the occurrence of anomalous experiences would be greatest in a worry induction group and least in a worry reduction condition. In order to enhance the clinical relevance of the study, we chose to study a bout of worry in patients with persecutory delusions in which worry was identified as at a high level; the effects of worry were studied in those who were prone to adopt this cognitive style.

## 2 Method

### 2.1 Participants

67 patients with persecutory delusions completed the study during the baseline assessment, prior to randomisation, of a clinical trial (ISRCTN23197625) (Freeman et al., 2012a). The participants were recruited from two mental health NHS Trusts: Oxford Health NHS Foundation Trust, Southern Health NHS Foundation Trust. The inclusion criteria were: a current persecutory delusion as defined by Freeman and Garety (2000); scoring at least 3 on the conviction scale of the PSYRATS delusions scale (i.e. at least 50% conviction in the delusion) (Haddock et al., 1999); that the delusion had persisted for at least three months; a clinical diagnosis of schizophrenia, schizoaffective disorder or delusional disorder; a clinically significant level of worry, as indicated by scores above 44 on the Penn State Worry Questionnaire (see Startup and Erickson, 2006); aged between 18 and 65; and no changes to medication in the past month. Criteria for exclusion were: a primary diagnosis of alcohol or substance dependency or personality disorder; organic syndrome or learning disability; a command of spoken English inadequate for engaging in therapy; and currently having individual CBT.

### 2.2 Baseline assessments

#### 2.2.1 Psychotic Symptom Rating Scales – delusions (PSYRATS; Haddock et al., 1999)

The PSYRATS – delusions is a six item multidimensional measure. It assesses the conviction, preoccupation, distress and disruption associated with delusions. Symptoms over the last week are rated. Higher scores indicate greater severity.

#### 2.2.2 Positive and Negative Syndrome Scale (PANSS; Kay, 1991)

The PANSS is a 30-item rating instrument developed for the assessment of patients with schizophrenia. Symptoms over the last week were rated (i.e. currently present). Higher scores indicate the greater presence of psychiatric symptoms.

#### 2.2.3 Green et al. Paranoid Thoughts Scale (GPTS; Green et al., 2008)

The GPTS is a thirty-two item measure of paranoid thinking. Part A assesses ideas of reference (e.g. ‘It was hard to stop thinking about people talking about me behind my back’) and Part B assesses ideas of persecution (e.g. ‘I was convinced there was a conspiracy against me’). Each item is rated on a 5-point scale. Higher scores indicate greater levels of paranoid thinking. The scale was completed for the period of the previous fortnight.

#### 2.2.4 Penn State Worry Questionnaire (Meyer et al., 1990)

The PSWQ is the most established measure of trait worry style and has been used in non-clinical and clinical populations (see review by Startup and Erickson, 2006). Each of the sixteen items is rated on a 5-point scale. Higher scores indicate a greater tendency to worry.

#### 2.2.5 Wechsler Abbreviated Scale of Intelligence (WASI; Wechsler, 1999)

The WASI is a standardised short and reliable measure of intelligence. The Vocabulary and Matrix Reasoning subtests were used to obtain an estimate of IQ.

### 2.3 Tasks completed before and after the randomisation condition

#### 2.3.1 Visual analogue rating scales (VAS)

In order to test the effects of the three conditions on worry and mood state, the participants marked on three 0 (‘Not at all’) to 100 (‘totally’) visual analogue scales the degree to which ‘right now’ they felt worried, anxious, or happy.

#### 2.3.2 Working memory: tasks from the Wechsler Adult Intelligence Scale III (WAIS-III) (Wechsler, 1997)

Three working memory tasks were used: digit span forwards, digit span backwards, and letter–number sequencing. These are the most commonly used working memory tasks in schizophrenia research (see Nuechterlein et al., 2004).

#### 2.3.3 Jumping to Conclusions (JTC): the beads task (Garety et al., 2005)

Data-gathering was assessed with a probabilistic reasoning task that has been extensively used with people with delusions. Participants are asked to request as many pieces of evidence (coloured beads) as they would like before making a decision (from which of two hidden jars the beads are drawn). The two jars have beads of two different colours in opposite ratios. The ratio of beads for the version used in the current study was 60:40. The colours of the beads in the jars were altered for the repeat of the task. The key variable is the number of beads requested before making a decision.

#### 2.3.4 Anomalous experiences I: Cardiff Anomalous Perceptions Scale (CAPS; Bell et al., 2006)

In the current study the CAPS was adapted into a state measure by asking whether any of the perceptual anomalies had occurred in the past 5 min, with participants simply responding with a Yes or No. A higher score represents the reporting of a greater number of perceptual anomalies. The scale covers a variety of anomalies including changes in levels of sensory intensity (e.g. ‘Are sounds much louder than they normally would be?’), distortion of own body or the external world (e.g. ‘Have you found the appearance of things or people seeming to change in a puzzling way e.g. distorted shapes or sizes or colour?’), sensory flooding (e.g. ‘Have you found that sensations happened all at once and flooded you with information?’), temporal lobe experiences (e.g. ‘Have you had the feeling that of being uplifted, as if driving or rolling over a road while sitting quietly?’) and hallucinations (e.g. ‘Have you heard noises or sounds when there was nothing about to explain them?’). In the current study we analysed the total score, but also tested separately the hallucination items and the non-hallucination perceptual anomalies.

#### 2.3.5 Anomalous experiences II: Cambridge Depersonalisation Scale (CDS; Sierra and Berrios, 2000)

We included a second scale to help capture the wide variety in anomalous experiences. The CDS was based on the view that ‘depersonalisation constitutes a syndrome which, in addition to ineffable feelings of ‘unreality’, also includes emotional numbing, heightened self-observation, changes in body experience, distortions in the experiencing of time and space, changes in the feeling of agency, feelings of having the mind empty of thoughts, memories and/or images, and an inability to focus and sustain attention’ (Sierra and Berrios, 2000). Two studies have shown that the scale assesses five distinct types of anomalous experiences (Sierra et al., 2005, Simeon et al., 2008). We used an adapted state version by asking about the occurrence of such experiences in the past few minutes. We used 19 items from the CDS, since ten items were not suitable for a state version (e.g. ‘previously familiar places look unfamiliar, as if I had never seen them before’). Higher scores indicate greater occurrence of anomalies. We examined the total score but also used four factors from the largest study of the CDS structure (Simeon et al., 2008): unreality of self (e.g. ‘Familiar voices (including my own) sound remote and unreal’), perceptual alterations (e.g. ‘I have the feeling that my hands or my feet have become larger or smaller’), unreality of surroundings (e.g. ‘What I see looks ‘flat’ or ‘lifeless’, as if I were looking at a picture’), and temporal disintegration (e.g. ‘It seems as if things that I have done had taken place a long time ago.’).

### 2.4 Procedure

The study was approved by an NHS research ethics committee. Participants completed the baseline assessments and the tasks assessing working memory, jumping to conclusions, and anomalous experiences. They then either received a worry induction, worry reduction, or a neutral condition. Randomisation to condition was carried out using www.randomisation.com. The tasks assessing psychotic processes were then repeated. The worry induction procedure was designed to encourage worry about topics that each participant already spent considerable time thinking about. It consisted of three stages. In the first stage the Worry Domains Questionnaire (WDQ; Tallis et al., 1992) was used to elicit common worries (that are not paranoid). In the second stage, participants took their two main worries identified from the WDQ and completed the catastrophising procedure for each (Vasey and Borkovec, 1992). The catastrophising procedure produces a ‘worry chain’ by the experimenter repeatedly responding to answers with ‘What is it that worries you about X?’ Participants were led to produce at least ten catastrophising steps for each worry. The third stage was 5 min of further engagement with worry, using instructions from McLaughlin et al. (2007): “*During this period we would like you to engage with your worrisome thoughts. Please refer to your list of worrisome topics. When you are asked to begin, please close your eyes and worry about your most worrisome topic in the way that you usually worry about it, but as intensely as you can, until the experimenter asks you to stop and open your eyes. If you normally worry about one topic at a time, please try to do the same during this period. However, if your thoughts change to another worry topic during this period feel free to allow these thoughts to continue. It is alright to change topics during this period if the changes occur naturally during the worry process.”* We have previously shown in non-clinical individuals that worry is increased using this procedure (Southgate, 2009). The worry reduction procedure consisted of a 10 min mindfulness relaxation exercise (‘Mindfulness of the breath’) (Kabat-Zinn, 2006), which could be accompanied by positive music if the participant chose (Delibes: Coppélia Act 1 Number 3 Mazurka) (Mayer et al., 1995). The neutral condition consisted of the person simply reading from a selection of magazines for ten minutes. Participants who received the worry induction condition also received if they wished the worry reduction condition at the end of testing.

### 2.5 Analysis

All analyses were carried out using SPSS Version 20 (IBM, 2011). To test change after the randomisation condition, analysis of covariance was used, with post-randomisation score as the dependent variable, group as a fixed factor, and baseline score as the covariate. When there was a main effect of group, least significant difference pairwise comparisons for the estimated marginal means were tested. All hypothesis testing was two-tailed.

## 3 Results

### 3.1 Demographic and clinical information

Basic demographic and clinical information for each of the randomisation groups is presented in Table 1. The participants predominately had clinical diagnoses of schizophrenia, were unemployed, and were prescribed anti-psychotic medication. They had levels of paranoia as assessed by the GPTS comparable to other studies of patients with persecutory delusions (e.g. Freeman et al., 2010) and levels of worry as assessed by the PSWQ comparable to patients with generalised anxiety disorder (e.g. Behar et al., 2003).

**Table 1:** Basic demographic and clinical information.

	Worry induction (*n* = 20)	Neutral condition (*n* = 22)	Worry reduction (*n* = 25)
Mean age in years (SD)	39.9 (12.2)	42.2 (11.6)	43.5 (11.3)
Sex:			
Male	11	13	12
Female	9	9	13
Ethnicity:			
White	19	20	24
Black Caribbean	0	0	1
Black African	0	0	0
Black Other	0	0	0
Indian	0	1	0
Pakistani	0	0	0
Other	1	1	0
Unemployed	15	14	15
Mean IQ (SD)	104.8 (17.6)	96.2 (19.4)	103.9 (17.3)
Diagnosis:			
Schizophrenia	17	16	15
Schizo-affective disorder	1	1	3
Delusional disorder	1	3	4
Psychosis NOS	1	3	3
Medication (Chlorpromazine equiv.):			
None	1	1	2
Low (1–200 mg)	2	5	5
Medium (200–400 mg)	4	7	10
High (≥400 mg)	13	9	8
Mean number of admissions in past 5 years (SD)	0.8 (1.3)	0.5 (0.7)	0.5 (1.0)
Mean PSYRATS delusions score (SD)	19.5 (1.8)	19.2 (2.7)	18.3 (3.0)
Mean PSYRATS delusions conviction (SD)	3.5 (0.5)	3.6 (0.5)	3.3 (0.7)
Mean PANSS score (SD)	84.6 (11.8)	82.4 (14.8)	77.5 (15.4)
Mean GPTS – Part A score (SD)	53.7 (14.3)	54.6 (14.7)	51.7 (16.8)
Mean GPTS – Part B score (SD)	61.1 (12.6)	58.9 (16.1)	54.8 (18.9)
Mean PSWQ score (SD)	66.1 (10.1)	66.1 (14.6)	64.9 (9.0)

### 3.2 The effects of the randomisation conditions

It can be seen in Table 2 that the worry induction condition significantly increased levels of worry compared with the other two conditions. The worry reduction condition reduced levels of worry. The worry induction condition also produced an increase in anxiety and a decrease in happiness compared with the other two conditions. The worry reduction condition did not significantly alter levels of anxiety or happiness.

**Table 2:** Estimated post-randomisation scores on the VAS scales (adjusted for baseline scores).

	Worry induction (*n* = 20)		Neutral condition (*n* = 22)		Worry reduction (*n* = 25)		Test of group effect	
	Mean	SE	Mean	SE	Mean	SE	*F* (2, 65)	*p*-Value
Worry	67.2^a^	4.3	44.2^b^	4.1	31.6^c^	3.8	19.5	<.001
Anxiety	65.8^a^	4.0	41.5^b^	3.8	34.1^b^	3.6	18.5	<.001
Happiness	29.2^a^	3.9	46.0^b^	3.8	45.5^b^	3.6	6.1	.004

Significant group differences (*p* < .05) are denoted by differing superscript letters.

### 3.3 Effects on psychotic processes

The post-randomisation performance of each group on the tasks assessing psychotic processes is summarized in Table 3. It can be seen that there are no differences between the groups in performance on working memory or jumping to conclusions tasks (controlling for baseline scores). However there are increases in the occurrence of anomalies of experience in the worry induction condition compared with the other two conditions. The only exception for anomalous experiences is that the occurrence of hallucinations is not affected by randomisation condition. (The same significant effects are found if PANSS total score is added as a covariate.)

**Table 3:** Estimated post-randomisation scores on the psychotic processes (adjusted for baseline scores).

	Worry induction (*n* = 20)		Neutral condition (*n* = 22)		Worry reduction (*n* = 25)		Test of group effect	
	Mean	SE	Mean	SE	Mean	SE	*F* (2, 65)	*p*-Value
Working memory								
Forward digit span	9.0	0.5	9.6	0.5	9.6	0.4	0.5	.618
Backward digit span	6.2	0.4	5.8	0.3	5.8	0.3	0.5	.637
Letter-number	8.2	0.4	8.7	0.4	8.4	0.3	0.6	.563

Jumping to conclusions								
Number of beads	6.4	0.6	6.5	0.6	7.8	0.6	1.8	.175

Anomalous experiences								
CAPS	4.7	0.5	4.3	0.5	3.3	0.5	2.3	.107
Hallucinations	1.2	0.2	1.1	0.2	1.3	0.2	0.3	.777
Non-hallucination anomalies	2.2^a^	0.3	1.9^a^	0.3	0.9^b^	0.3	5.5	.006

CDS	5.0^a^	0.5	2.5^b^	0.5	2.4^b^	0.4	10.0	<.001
Unreality	1.7^a^	0.2	1.1^a,b^	0.2	0.7^b^	0.2	4.8	.012
Perceptual	0.9^a^	0.1	0.4^b^	0.1	0.2^b^	0.1	6.3	.003
Surroundings	0.5^a^	0.1	0.2^b^	0.1	0.2^b^	0.1	3.8	.028
Temporal	1.1^a^	0.1	0.6^b^	0.1	0.5^b^	0.1	4.5	.015

Significant group differences (*p* < .05) are denoted by differing superscript letters.

## 4 Discussion

Patients with persecutory delusions who worried were the focus of this study. Previous research has shown that this is the most common presentation of paranoia (e.g. Freeman and Garety, 1999, Morrison and Wells, 2007, Freeman et al., 2010). We examined the effects of a bout of worry in these individuals on processes customarily considered as markers of psychosis. Given that worry was known to occur frequently in these patients we wanted to know whether it impacted on psychotic processes. The results were very clear. The occurrence of worry did not affect working memory or jumping to conclusions, but it did lead to an increase in a range of low-level anomalous experiences. Increases in sensations of the unreality of self and surroundings (e.g. feeling of being a detached observer), perceptual alterations (e.g. body feeling very light as it if were floating), and temporal disintegration (e.g. seeming as if things done recently had taken place a long time ago) were all more likely to occur after a period of worry. Worrying less reduced the occurrence of anomalies of sensory intensity (e.g. lights or colours seeming brighter or more intense than usual) and sensory flooding (e.g. difficulty distinguishing one sensation from another). Our assessment covered a wide variety of anomalous experiences and it was only the occurrence of hallucinations that was unaffected. The point of interest learned from the experiment is that a period of worry in these patients was creating a range of potentially confusing and puzzling perceptual experiences that are known to predict paranoid thoughts. Worry was causing odd subjective states that are known to lead to odd ideas. This is an illustration of an interaction of affective with psychotic processes, and adds to our understanding of how worry may contribute to delusional experience. This information can be incorporated into the emerging therapies that target worry in psychosis (Foster et al., 2010, Hepworth et al., 2011); clinicians can assess for and normalise the occurrence of such anomalies after a period of worry in patients with persecutory delusions.

Working memory and jumping to conclusions were not altered by a period of worry. This fails to replicate the finding of worry impeding working memory in high non-clinical worriers (e.g. Leigh and Hirsch, 2011). However these previous studies assessed working memory and worry concurrently, while in the current study we simply assessed the effects after a period of encouraging worry. We do not know the extent to which individuals in the worry induction condition were continuing to worry, which is a limitation of the study. It is also clear that the worry induction was having effects more generally on mood; this is to be expected but limits the precision with which the occurrence of anomalous experiences can be attributed specifically to worry. Previous studies have had mixed findings on jumping to conclusions when manipulating levels of anxiety (So et al., 2008, Lincoln et al., 2010a), and the current study does not support the idea that JTC changes with anxious mood state. We examined three key processes and did not alter significance levels for multiple testing, agreeing with the view that ‘simply describing what tests of significance have been performed, and why, is generally the best method of dealing with multiple comparisons’ (Perneger, 1998). The final key limitation is that we did not assess levels of paranoia before and after each randomisation condition which means that we could not test whether and how paranoia increased after worrying; we simply tested the effects of worry on psychotic processes, and a larger experimental study that can examine mediation is now warranted (Emsley et al., 2010). Our contention is that the role of worry will receive increasing attention in the study and treatment of delusions.

## Role of the funding source

The study was supported by a grant (09/160/06) from the Efficacy and Mechanism Evaluation (EME) Programme, which is funded by the UK Medical Research Council and UK NHS National Institute for Health Research. This study was included in the grant application for the clinical trial. The funder has not altered or reviewed this paper.

## Contributors

The study was designed by Daniel Freeman, Helen Startup, Graham Dunn and David Kingdon. Daniel Freeman drafted the paper, which was commented upon by all authors. Emma Černis and Gail Wingham collected the data. Daniel Freeman and Graham Dunn carried out the analysis.

## Conflict of interest

None.
